# Open Access Publishing Metrics, Cost, and Impact in Health Professions Education Journals

**DOI:** 10.1001/jamanetworkopen.2024.39932

**Published:** 2024-10-16

**Authors:** Sawsan Abdel-Razig, Dora Stadler, Leen Oyoun Alsoud, Sophia Archuleta, Halah Ibrahim

**Affiliations:** 1 Department of Academics, Cleveland Clinic Abu Dhabi, Abu Dhabi, United Arab Emirates; 2 Department of Internal Medicine, Cleveland Clinic Lerner College of Medicine, Case Western Reserve University, Cleveland, Ohio; 3 Department of Medicine, Weill Cornell Medicine-Qatar, Doha, Qatar; 4 Department of Medical Science, Khalifa University College of Medicine and Health Sciences, Abu Dhabi, United Arab Emirates; 5 Department of Medicine, Yong Loo Lin School of Medicine, National University of Singapore, Singapore; 6 Division of Infectious Diseases, Department of Medicine, National University Hospital, National University Health System, Singapore

## Abstract

**Importance:**

Publishing in health professions education (HPE) journals is an integral component of academic discourse and career progression. Research in this field is shifting to an open access (OA) publishing model.

**Objective:**

To identify the characteristics and publishing models of HPE journals and explore potential associations between publication costs and journal metrics.

**Design, Setting, and Participants:**

This cross-sectional study was conducted between September 20, 2023, and February 14, 2024, using the World Bank purchasing power parity (PPP) index to analyze relative costs of article processing charges (APCs). Data on journal characteristics, impact metrics, APCs, and waiver or discount were extracted from the National Library of Medicine, Scimago, Scopus, journal websites, and email correspondence with editorial staff of journals. All HPE journals indexed in PubMed, written in or translated into English, and with HPE as a core component of their mission were included in the analysis.

**Main Outcomes and Measures:**

Two-year impact factor, H-index, cite score, Scientific Journal Ranking, and APC.

**Results:**

Among the 51 journals included, 27 (53%) adopted OA-only and 24 (47%) adopted hybrid publishing models. The median (IQR) APC for all journals was $2820.00 ($928.00-$3300.00). Associations were observed between impact factor and APC (β coefficient, $386.84; 95% CI, $226.84-$546.84; *P* < .001) and between cite score and APC (β coefficient, $282.40; 95% CI, $148.12-$416.61; *P* < .001). Of 20 journal websites with information regarding fee waivers or discounts, 7 journals (35%) confirmed fee waiver or discount. The PPP index analysis of the top 39 countries publishing HPE research showed that the financial burden of meeting the median APC for publication was 1.94 to 10.26 times higher for authors from lower-income countries than for authors from the US.

**Conclusions and Relevance:**

Results of this cross-sectional study suggest that adoption by HPE journals of an OA publishing model was high but access to APC waivers or discounts was limited. These factors create barriers to equitable OA practices, necessitating concerted efforts, such as increasing transparency of publishing costs, implementing economic impact analysis, expanding waivers to eligible authors, and applying holistic impact factor scoring.

## Key Points

**Question:**

What are the characteristics and publishing models of health professions education (HPE) journals, and what are the financial implications of open access (OA) publishing?

**Findings:**

In this cross-sectional study of 51 HPE journals, over half of the journals adopted OA publishing models and only a few confirmed providing article processing charge (APC) waivers or discounts. The relative APC for authors from lower-income countries ranged from 1.94 to 10.26 times the cost for authors based in the US.

**Meaning:**

Findings of this study suggest that barriers to equitable OA practices exist among HPE journals and should be addressed to promote equity in authorship and research dissemination.

## Teaser

This cross-sectional study examines characteristics and publishing models of journals in health professions education research and assesses the implications of open access publishing.

## Introduction

Spurred by the emergence of the internet in the 1990s, open access (OA) publishing has reshaped the way that biomedical literature is accessed and disseminated. Worldwide, there is increasing recognition that research findings and scholarly efforts should be freely accessible to all.^1^ The 2001 inaugural conference of the Budapest Open Access Initiative—a collective of researchers, publishers, foundations, universities, and societies—was a pivotal event in the OA movement and defined OA in peer-reviewed literature as “the free availability [of literature] on the public internet, permitting any user to read, download, copy, distribute, print, search, or link to full texts of articles.”^2^ The most conformant to the Budapest Open Access Initiative definition is the gold OA, whereby journals make all of their articles available online for free after publication and are thus identified in the Directory of Open Access Journals. Current OA publishing models offer varying levels of article access, with defined subtypes and differing levels of restriction.

Although the number of OA publications continues to increase exponentially, the model is not without controversy, including costly article processing charges (APCs), OA citation advantage, and the potential to facilitate paper mills and predatory journal practices.^3^ Overall, OA publishers increasingly rely on pay-to-publish models through APCs, which are paid by authors or their funders or institutions. These fees have been observed across biomedical research disciplines^4,5,6^ and are often substantial.^7^ Some journals have APC waiver policies, but studies have shown variability in policy and implementation,^8^ making it difficult to assess the effectiveness of these waivers.

Similar to biomedical scholarship, health professions education (HPE) research is undergoing a shift to OA publishing.^9^ Most HPE research is unfunded, thereby increasing the financial considerations for authors when selecting the journals in which to disseminate their research.^10^ The objective of the present study was to identify the characteristics and publishing models of HPE journals and explore potential associations between publication costs and journal metrics.

## Methods

Between September 20, 2023, and February 14, 2024, we conducted an internet-based cross-sectional study of publicly available websites, which we augmented with direct email correspondence with journals that advertised fee waivers, to identify characteristics and fees related to OA publishing in HPE. We converted all costs to US dollars on February 14, 2024, based on the currency conversion rates at that time. The Cleveland Clinic Abu Dhabi Institutional Review Board deemed this study exempt from ethics review and waived the informed consent requirement because it was not considered human participant research. We followed the Strengthening the Reporting of Observational Studies in Epidemiology (STROBE) reporting guideline.^11^

### Conceptual Framework 

We used an economic impact assessment framework to assess the financial implications of publishing in OA journals for authors from different countries.^12^ The World Bank purchasing power parity (PPP) index^13^ was used to better understand the relative cost of APCs. The PPP index is a ratio of the monetary units required to purchase the same good (in this analysis, the cost of an OA article) in different countries.^13^ Compared with a simple currency conversion, the PPP index enables the calculation of the relative difference in the burden of cost between authors from different nations.

### Eligibility Criteria

**Figure 1.**
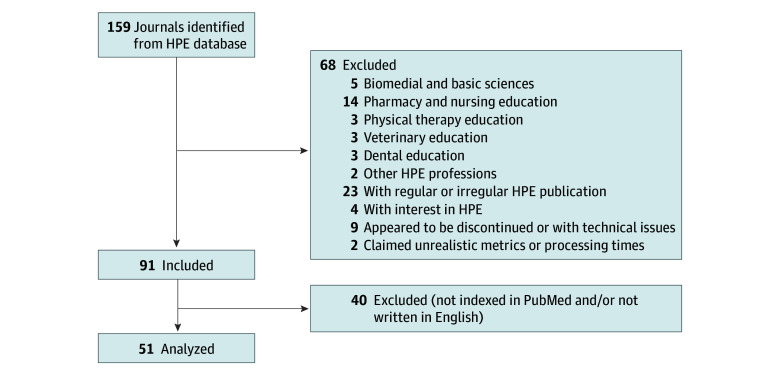
Flow Diagram of Journal Inclusion and Exclusion HPE indicates health professions education.

A comprehensive database of HPE journals is maintained^14^ and updated annually, with the last update performed in May 2023. This database is an accepted resource for medical educators globally and served as the starting list for the present study. The list was compared for accuracy and completion against the Annotated Bibliography of Journals for Educational Scholarship developed by the Association of American Medical Colleges^15^ and the Medical Education Journal List-24, a seed set of HPE journals used by researchers as a bibliometric proxy for the field of medical education.^16^ We included all PubMed-indexed journals derived from these databases, written in or translated into English, that explicitly have HPE as a core component of their mission. General journals that only occasionally publish HPE research and journals that were no longer in publication were excluded (Figure 1). All included journals were screened using Cabells Scholarly Analytics.^17,18^

### Data Collection 

Using a standardized data collection template, 2 researchers (L.O.A., S.A.-R.) independently collected data through a comprehensive search of publicly available sources. Discrepancies were discussed with the entire research team and resolved by consensus. An audit of a random 20% sample of the data was also performed by one of us (H.I.) to ensure data accuracy and reproducibility. Journals with fee waiver or discount information on their websites were contacted via their listed email address. The full list of included journals is provided in eTable 1 in Supplement 1.

### Data Sources

Internet searches of publicly accessible websites were conducted to simulate the information a prospective author would be able to find. Data were extracted from the National Library of Medicine catalog,^19^ Scimago,^20^ Scopus,^21^ and each journal’s website. H-index, which refers to the number of papers published by the journal and the number of citations to those papers,^22^ was obtained from Scimago. Impact factor, APC, and fee waiver details were collected from individual journal websites. Cite score, which is the mean number of citations received per document over a 4-year period, and Scientific Journal Ranking, which is the rank of journals within a specific research field based on various metrics,^22^ were obtained from Scopus. Journals that are not tracked in the Journal Citation Reports database and, thus, do not have impact factors were assigned an impact factor of 0. The country of publication, year of publication, and publisher name were obtained from the National Library of Medicine catalog and Scopus.

### Variables Extracted

The final list of items extracted for each journal included journal name, website, publisher name, contact email, year of initial publication, country of publication, publication model (subscription, hybrid, or OA only), most recent 2-year impact factor, most recent H-index, Scientific Journal Ranking, APC, and currency. We also included submission fees (when relevant) and the availability of fast-track and discount waiver policies. Publishing models were defined as gold OA only, hybrid, or traditional subscription.

### Statistical Analysis

We used R, version 3.65 (R Project for Statistical Computing) to conduct statistical analysis. Medians and IQRs were calculated. Linear regression models were used, with APC as the dependent variable and with cite score and impact factor as the independent variables. Two-sided *P* < .05 indicated statistical significance.

Income statuses of the 39 highest-ranked nations in HPE research output^23^ were classified per the World Bank categories: low income, lower middle income, upper middle income, and high income.^24^ Equivalent relative cost was ascertained using the PPP index.^13^ For the purpose of this comparative analysis, the expense factor for the US was set as 1.00.

## Results

We identified 159 HPE journals, of which 51 (32%) met the study inclusion and exclusion criteria (Figure 1). Table 1 lists journal characteristics. None of the journals exclusively used a traditional subscription-based publishing model. Approximately half of the journals had a gold OA-only (27 [53%]) or hybrid (24 [47%]) publishing model. The majority of journals were based in North America (25 [49%]) and Europe (17 [33%]). Eighteen journals (35%) were classified within quartile 1, of which 8 were published in North America, 8 in Europe, and 2 in Asia.

**Table 1.** Journal Characteristics, Publishing Models, and Journal Metrics

	Journals, No. (%)		
	All (N = 51)	Gold OA only (n = 27)	Hybrid (n = 24)
Year of establishment	1905-2020	1981-2020	1905-2017
Region			
North America	25 (49)	11 (41)	14 (58)
Europe	17 (33)	7 (26)	10 (42)
Asia	7 (14)	7 (26)	0
Oceania	1 (2)	1 (4)	0
Africa	1 (2)	1 (4)	0
APC present			
Yes	43 (84)	19 (70)	24 (100)
No	8 (16)	8 (30)	0
Quartile			
1	18 (35)	6 (22)	12 (50)
2	19 (37)	10 (37)	9 (38)
3	6 (12)	3 (11)	3 (12)
Unclassified	8 (16)	8 (30)	0
Impact factor, median (IQR)	1.80 (0.00-3.45)	0.50 (0.00-3.05)	2.50 (1.68-4.10)
Cite score, median (IQR)	2.90 (2.00-4.70)	2.20 (0.00-3.25)	3.75 (2.63-5.88)
H-index, median (IQR)^a^	33.00 (15.00-68.50)	17.00 (0.00-35.50)	60.00 (30.25-111.50)
SJR, median (IQR)	0.54 (0.31-0.92)	0.47 (0.00-0.60)	0.77 (0.52-1.16)
APC, median (IQR), $	2820.00 (928.00-3300.00)^b^	1000.00 (0.00-1972.50)^b^	3309.50 (3127.50-3665.00)
APC excluding $0 fee			
No. (%)	43 (84)	19 (70)	NA
Median (IQR), $	3051.00 (1955.00-3404.50)^c^	1725.00 (928.00-2565.00)^c^	3309.50 (3127.50-3665.00)

Abbreviations: APC, article processing charge; NA, not applicable; OA, open access; SJR, Scientific Journal Ranking.

^a^ H-index reflects the number of papers published by the journal and number of citations to those papers.

^b^ Includes all journals.

^c^ Excludes journals with no APC.

Among all journals, 15 (29%) did not have an impact factor, and 9 (18%) did not have a cite score. The overall median (IQR) impact factor was 1.80 (0.00-3.45), and the median (IQR) cite score was 2.90 (2.00-4.70). The top-ranked hybrid journal was the *British Journal of Anaesthesia*, with a cite score of 14.90 and an impact factor of 9.80. *Medical Education Online* was the top-ranked gold OA-only journal, with a cite score of 5.70 and an impact factor of 4.60.

### Article Processing Charges

Eight of 51 journals (16%) did not charge fees for OA publication. The median (IQR) APC for all journals was $2820.00 ($928.00-$3300.00). Ten journals (20%) had an APC greater than $3500.00. When the 8 journals without an APC were excluded, the median (IQR) APC was $3051.00 ($1955.00-$3404.50). The median (IQR) APCs were $1000.00 ($0.00-$1972.50) for gold OA-only journals and $3309.50 ($3127.50-$3665.00) for hybrid journals.

**Figure 2.**
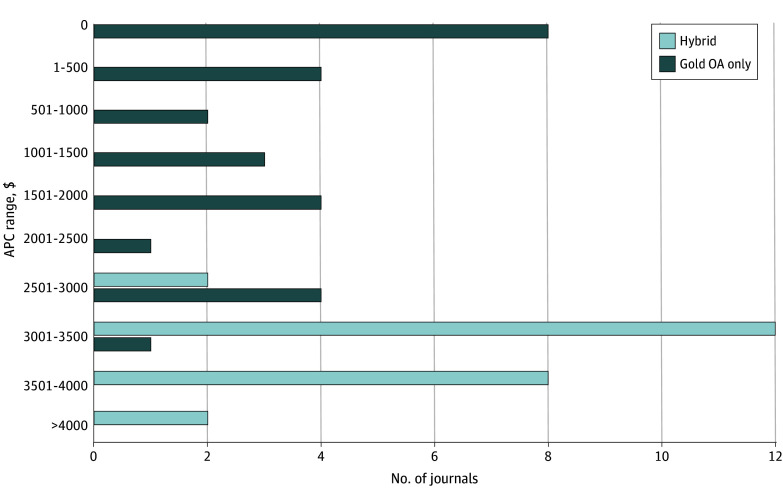
Article Processing Charge (APC) Range Distributions The APC range distributions were compared between hybrid (n = 24) and gold open access (OA)–only journals (n = 27).

Linear regression analysis revealed an association between impact factor and APC (β coefficient, $386.84; 95% CI, $226.84-$546.84; *P* < .001) and between cite score and APC (β coefficient, $282.40; 95% CI, $148.12-$416.61; *P* < .001). Figure 2 depicts the APC range distribution for all journals. We observed APCs of less than $2500.00 exclusively in gold OA-only journals (n = 27), whereas APCs of more than $3300.00 were observed in hybrid journals (n = 24).

### Publisher Data

Twenty-four publishers were identified (eTable 2 in Supplement 1). Publishers with the highest APCs were Wiley, with a median (IQR) APC of $3620.00 ($3380.00-$3665.00); Wolters Kluwer, with a median (IQR) APC of $3578.00 ($979.75-$4460.25); Taylor & Francis, with a median (IQR) APC of $3300.00 ($3300.00-$3300.00); Frontiers Media, with a median (IQR) APC of $3295.00 ($3295.00-$3295.00); Springer, with a median (IQR) APC of $3090.00 ($2940.00-$3390.00); and Elsevier, with a median (IQR) APC of $3045.50 ($2955.00-$3545.00). Of these publishers, 19 (79%) exclusively offered the gold OA publication model, with 5 (26%) offering OA publication without APC. Among the exclusive gold OA publishers, Frontiers Media had the highest median (IQR) APC at $3295 ($3295.00-$3295.00).

### PPP Index

Setting an expense factor of 1.00 for the US, we used the PPP index to compare the relative cost of the median (IQR) APC of $2820.00 for authors from the top 39 most prolific nations in HPE research ranked in order of their output.^23^ For example, the median APC of $2820.00 for an author from Egypt represents an equivalent cost of $28 935.26 for US authors (Table 2). There were 26 high-income (67%), 12 upper- or lower middle–income (31%), and 0 low-income countries, with 1 (3%) without World Bank data (Table 2). Authors from lower- or upper middle–income countries had a relative cost to publish in HPE journals that ranged from 1.94 to 10.26 times higher than the range of 0.87 to 2.16 for authors from high-income countries except in Iran, which had an expense index of 0.46.

**Table 2.** Purchasing Power Parity to Identify Median Article Processing Charge and Relative Cost

HPE research output rank	Country of publication	Country’s income status	Relative cost, $	Expense factor
1-5	US	High income	2820.00	1.00
	UK	High income	3156.47	1.12
	Canada	High income	3225.73	1.14
	Australia	High income	3064.67	1.09
	Germany	High income	3689.40	1.31
6-10	Netherlands	High income	3688.96	1.31
	India	Lower middle income	11 719.88	4.16
	France	High income	3688.96	1.31
	Brazil	Upper middle income	6471.43	2.29
	Italy	High income	4304.63	1.53
11-20	Saudi Arabia	High income	5292.60	1.88
	China	Upper middle income	5614.38	1.99
	Spain	High income	4304.55	1.53
	Japan	High income	4366.07	1.55
	New Zealand	High income	3131.07	1.11
	South Korea	High income	4809.73	1.71
	Ireland	High income	3228.44	1.14
	Denmark	High income	3159.37	1.12
	Switzerland	High income	2439.30	0.87
	Taiwan	No data available^a^	No data available^a^	No data available^a^
21-39	Egypt	Lower middle income	28 935.26	10.26
	Mexico	Upper middle income	5476.06	1.94
	South Africa	Upper middle income	7079.59	2.51
	Thailand	Upper middle income	9285.31	3.29
	Argentina	Upper middle income	19 073.18	6.76
	Portugal	High income	5164.02	1.83
	Turkey	Upper middle income	13 489.83	4.78
	Norway	High income	3425.94	1.21
	Sweden	High income	3488.04	1.24
	Poland	High income	5867.66	2.08
	Malaysia	Upper middle income	8898.11	3.16
	Chile	High income	6097.45	2.16
	Belgium	High income	3688.76	1.31
	Austria	High income	3689.00	1.31
	Iran	Lower middle income	1303.24	0.46
	Pakistan	Lower middle income	13 962.26	4.95
	Israel	High income	2918.27	1.03
	Greece	High income	5164.54	1.83
	Singapore	High income	4665.56	1.65

Abbreviation: HPE, health professions education.

^a^ No data from the World Bank.

### Fee Waivers or Discounts

Twenty of the 51 journal websites (39%) included information regarding fee waivers or discounts. More than half of these journals had a gold OA-only publishing model (11 of 20 [55%]). Two journals (10%) required input of specific manuscript details before confirming eligibility for a fee waiver; these journals were excluded. One journal (5%) did not respond to our inquiry. Seven journals (35%) confirmed providing discounts and/or waivers to mitigate APC fees for authors from low-income or lower middle–income countries. The remaining 10 journals (50%) stated that they did not have fee waivers or discounts available. Details about the waiver policy for these journals are provided in eTable 3 in Supplement 1.

## Discussion

In this cross-sectional analysis of OA publishing in HPE research, we found that all journals included OA publishing options, although notable differences exist between hybrid and gold OA-only journals. First, hybrid journals had significantly higher journal metrics, including impact factors, cite scores, and H-indices, and were more frequently ranked in quartile 1. Hybrid journals also had higher APCs for OA publishing. Moreover, journals from the leading academic publishers, including Wiley and Taylor & Francis, had the highest APCs.

While OA ideally enables increased access to published knowledge for scholars from low- and middle-income countries, APCs may limit these authors’ ability to disseminate new knowledge and deprive the larger scientific community of their work. Prior research suggests that unaffordability of APCs has led to substantial reductions in publications from scholars from low-income countries.^3^ The PPP index analysis revealed that the cost of OA publication can pose a substantial financial burden. Of the top 39 countries producing HPE research, 26 were classified as high income, with the rest being lower- and upper middle–income countries; there were no low-income countries represented in this group. Additionally, the PPP index analysis provided important insights into potential publication challenges. For authors from lower- or upper middle–income countries, the relative cost of the median APC required to publish in HPE research ranged from 1.94 to 10.26 compared with a range of 0.87 to 2.16 for authors from high-income countries. The only exception was Iran, with an expense index of 0.46, although this likely represented complex economic factors and potential limitations of the PPP index in the Iranian context. The PPP index represents relative financial burdens by providing information on the purchasing power of a given currency compared with the US dollar for the same product. These financial obstacles are not limited to authors from lower-income countries. Although recent studies show that funding is increasingly available for HPE research through affiliations with government, university, or private nonprofit organizations,^25^ authors who lack institutional or governmental funding are disadvantaged as OA publishing models are increasingly adopted.

The findings reveal an association of high cite scores and impact factors with increasing APCs. The highest fees were associated with hybrid journals, which include established journals with large readerships and big publishers that rely primarily on traditional subscription models. Since hybrid journals offered manuscript publication without fees, high APCs were imposed for the OA option. The OA publishing in these high-impact journals provides a citation advantage to authors who can afford the APCs. Specifically, authors without funding are less likely to benefit from the increased public engagement, social media dissemination, and improved alternative citation metrics of OA publications,^1^ a factor likely associated with the Global North (ie, countries with known economic and political power) predominance in HPE publications.^26,27^

Although fee discounts and waiver programs are intended to help mitigate financial barriers to OA publishing, we found limited information and a lack of transparency. Several journals advertised waivers or discounts on their websites, but such information was difficult to obtain and most, ultimately, did not offer these data. Moreover, none of the journals that confirmed fee waiver or discount opportunities had a hybrid publishing model. This finding suggests that publishers that profit from OA journals have placed the onus on authors and nonprofit organizations, such as funding agencies and institutions, to incur the costs of publishing services.

Transparency is needed regarding the costs of OA publishing given the reduced costs of online (vs print) publication and the continued use of voluntary peer reviewers. Journals should use a robust economic impact assessment when making decisions regarding publishing models. This assessment identifies the economic consequences of policies and the measures that help mitigate disparities in the dissemination of scholarship. Consistent with other studies,^28^ we found that most HPE research publishers were located in North America and Europe, with all but one quartile 1 journal published in these 2 regions of the Global North. It is interesting to note that journals from all other regions used gold OA-only publishing models, while hybrid journals were exclusively from North America or Europe. Previous studies have highlighted considerable geographic disparities in HPE research, with 80% of authorship originating from 4 high-income countries (namely, the US, the UK, Canada, and Australia),^23^ reflecting the greatest geographic disparity compared with several other fields, including biological sciences, medicine, and education.^23,29^

While the OA movement has improved access to information for HPE researchers in resource-poor settings, it has created an additional barrier that hinders authors’ ability to contribute to the literature. This barrier is especially worrisome as more journals move to OA-only publishing, with several instances of journals starting their OA publishing model without fees but later instituting high APCs.^30^ Ultimately, this situation is a disadvantage for the global HPE community because it restricts geographic and topical diversity, and the published landscape becomes increasingly limited to discourses from select high-income institutions and countries. Moreover, it may contribute to the spread of predatory publishing among authors in lower-income countries who can afford only lower APCs of illegitimate journals.^3,29^

Although there are many barriers to publication for authors in low-resource settings, potential solutions include offering more fee waivers or discounts for OA publishing and improving the transparency and commitment of journals with these programs. Hybrid journals also should offer fee waivers to eligible authors. Alternatively, instituting selective lifting of access or paywall restrictions may help facilitate the dissemination of knowledge generated in regions underrepresented in the HPE literature. Furthermore, including measures of equity of access in the journal’s impact factor, beyond the number of citations, is critical for equitable OA. For example, the geographic distribution of citations and authors can be considered when assessing global impact. Much like the ongoing movement in education to incorporate social mission rankings^31^ to better inform student selection,^32^ we call on platforms such as Web of Science and Clarivate to incorporate equity-related metrics that move the needle from impact factor to equity factor.

### Limitations

This study should be considered in the context of several limitations. First, we studied only English-language indexed literature. Additionally, the journals we analyzed do not represent all publishers of HPE research but were restricted to those dedicated to a medical education mission. Second, the PPP index we used may be outdated, as publicly available indices at the time of publication were updated to 2023, and currency devaluation and exchange rates in lower-income nations can fluctuate from year to year. Third, we emailed only journals that offered fee waivers. It is possible that other journals would have offered discounts if contacted.

## Conclusions

The discourse in HPE research continues to be dominated by authors and topics originating from high-income countries in the Global North. The OA movement has been touted to increase access and diversity in the medical literature. The findings of this cross-sectional study suggest that adoption by HPE journals of the OA publishing model was high, APCs were high and associated with higher impact factors, and access to waivers or discounts was limited. Substantial barriers to equitable OA existed, including journal reliance on costly APCs, the existence of restricted access to publications from the most widely cited journals in this field, and the variable availability of APC discount or waiver programs. Mechanisms to alleviate these impediments include increasing the transparency of publishing costs, implementing a robust economic impact analysis when instituting APCs, expanding waivers to eligible authors, and applying holistic impact factor scoring that considers the diversity of authorship and ease of access.

## Notes

Supplement 1. **eTable 1.** Health Professions Education Journal Characteristics **eTable 2.** Median Article Processing Charge Per Publisher **eTable 3.** Discount and Waiver Policies of Health Professions Education Journals

Supplement 2.
Data Sharing Statement
