# Supplementary material for: Open Access Publishing Metrics, Cost, and Impact in Health Professions Education Journals
Source: JAMA Netw Open. 2024 Oct 16;7(10):e2439932. doi: 10.1001/jamanetworkopen.2024.39932 (PMC11484459; doi:10.1001/jamanetworkopen.2024.39932)
Supplement: Supplement 1. — eTable 1. Health Professions Education Journal Characteristics eTable 2. Median Article Processing Charge Per Publisher eTable 3. Discount and Waiver Policies of Health Professions Education Journals [file jamanetwopen-e2439932-s001.pdf]

Supplemental Online Content

Abdel-Razig S, Stadler D, Oyoun Alsoud L, Archuleta S, Ibrahim H. Open access publishing metrics, cost, and impact in health professions education journals. *JAMA Netw Open*. 2024;7(10):e2439932. doi:10.1001/jamanetworkopen.2024.39932

eTable 1. Health Professions Education Journal Characteristics

eTable 2. Median Article Processing Charge Per Publisher

eTable 3. Discount and Waiver Policies of Health Professions Education Journals

This supplemental material has been provided by the authors to give readers additional information about their work.

eTable 1. Health Professions Education Journal Characteristics

Journal name	Year established (Pub start year)	Country of publication	Most recent impact factor	2022 Citescore	2022 h index	2022 SJR	Open access /APC fees	Amount in USD	Waiver/ discount /both/none
Academic Emergency Medicine	1994	North America	4.4	6.4	137	1.462	Yes	3,710	None
Academic Medicine	1989	North America	7.4	6.7	173	1.579	Yes	4,668	None
Academic Pathology	2014	North America	1	2.2	13	0.313	Yes	1,950	Both
Academic Pediatrics	2009	North America	3.1	3.9	87	1.135	Yes	3,000	None
Academic Psychiatry	1989	North America	2.5	3.1	53	0.68	Yes	3,490	None
Academic Radiology	1994	North America	4.8	7.2	105	1.064	Yes	3,510	None
Advances in Health Sciences Education	1996	Europe	4	5.7	75	1.2	Yes	3,090	None
Advances in Medical Education and Practice	2010	Oceania	2	2.9	36	0.505	Yes	2,680	No email was sent as a form was needed for evaluation
AEM Education and Training	2017	North America	1.8	2.2	20	0.552	Yes	3,140	None
African Journal of Health Professions Education	2009	Africa	0.5	0	0	0	Yes	438	None
AMEE MEdEdPublish	2016	Europe	0	0	0	0	Yes	1,960	None
American Journal of Surgery	1905	North America	3	4.6	163	0.85	Yes	3,650	None
ATS Scholar	2020	North America	0	2.1	0	0	Yes	1,346	None
BMC Advances in Simulation	2016	Europe	0	0	0	0	Yes	2,690	Both
BMC Medical Education	2001	Europe	3.6	4.5	87	0.914	Yes	2,890	Both
British Journal of Anaesthesia	1923	Europe	9.8	14.9	201	2.487	Yes	3,870	None
Canadian Medical Education Journal	2010	North America	0	0	0	0	No	0	None

© 2024 Abdel-Razig S et al. *JAMA Network Open*

Education for Health	1996	Asia	0.7	0	38	0.268	No	0	None
Education for Primary Care	2001	Europe	1.3	2.1	22	0.505	Yes	3,300	None
Family Medicine [Society of Teachers of Family Medicine] https://journals.stfm.org/familymedicine/	1981	North America	2.24	2.6	71	0.61	No	0	None
Frontiers in Medicine - Health Professions Education	2014	Europe	3.9	3.6	71	0.926	Yes	3,295	No email was sent as a form was needed for evaluation
Gerontology & Geriatrics Education	1980	Europe	1.6	3	28	0.591	Yes	3,300	None
GMS Journal for Medical Education	2016	Europe	0	2.9	25	0.47	Yes	856	None
International Journal of Medical Education	2010	Europe	0	2.8	33	0.538	Yes	1,725	Both
JMIR Medical Education	2015	North America	3.6	5	23	0.837	Yes	1,985	None
Journal of Advances in Medical Education and Professionalism [Iran]	2013	Asia	0	0.8	4	0.22	Yes	150	None
Journal of Cancer Education	1986	Europe	1.6	3.6	48	0.648	Yes	3,590	None
Journal of Continuing Education in the Health Professions	1988	North America	1.8	2.7	62	0.523	Yes	3,319	None
Journal of Education and Health Promotion [India]	2012	Asia	1.4	2.3	17	0.411	Yes	200	None
Journal of Educational Evaluation	2006	Asia	4.4	4.7	17	0.917	No	0	None

© 2024 Abdel-Razig S et al. *JAMA Network Open*

for Health Professions [Korea]									
Journal of Graduate Medical Education	2009	North America	0	2.1	34	0.502	No	0	None
Journal of Interdisciplinary Virtual Learning in Medical Sciences [Iran-based]	2016	Asia	0	0	0	0	No	0	None
Journal of Interprofessional Care	1992	Europe	2.7	4.5	83	0.904	Yes	3,300	None
Journal of Interprofessional Education and Practice	2015	North America	0	1.5	13	0.298	Yes	2,820	None
Journal of Medical Education and Curricular Development	2014	North America	2.8	0	0	0	Yes	2,500	Waiver
Journal of Research in Interprofessional Practice and Education	2009	North America	0	0	0	0	Yes	1,400	None
Journal of Surgical Education	2007	North America	2.9	5.1	66	0.935	Yes	3,040	None
Kaohsiung Journal of Medical Sciences [Taiwan-based]	1996	Asia	3.3	4.9	44	0.597	Yes	1,000	None
Korean Journal of Medical Education	2009	Asia	0	2.8	16	0.485	Yes	400	None
MedEdPORTAL	2005	North America	0	1.9	14	0.498	No	0	None
Medical Education	1976	Europe	7.1	7.5	155	1.629	Yes	5,250	None
Medical Education Online	1996	North America	4.6	5.7	43	1.07	Yes	2,630	Both
Medical Science Educator	2011	North America	1.7	1.6	20	0.393	Yes	3,090	None
Medical Teacher	1979	Europe	4.7	6.8	131	1.217	Yes	4,000	None

© 2024 Abdel-Razig S et al. *JAMA Network Open*

Morphologie	1997	Europe	0	2.1	23	0.3	Yes	3,051	None
Peer-Reviewed Reports in Medical Education Research(PRiMER)	2017	North America	0	0	0	0	No	0	None
Perspectives on Medical Education	2012	Europe	4.113	6.9	35	1.259	Yes	1,176	Waiver
Simulation in Health Care	2006	North America	2.4	3.4	58	0.596	Yes	3,837	None
Teaching and Learning in Medicine	1989	North America	2.5	4.7	55	1.145	Yes	3,300	None
The Clinical Supervisor	1983	North America	1.2	3.5	31	0.41	Yes	3,300	None
The Clinical Teacher	2004	Europe	1.8	2.4	31	0.433	Yes	3,620	None

© 2024 Abdel-Razig S et al. *JAMA Network Open*

eTable 2. Median of Article Processing Charge Per Publisher

Publisher	Number of journals	Median APC (USD)
1. Accreditation Council for Graduate Medical Education	Gold Open Access (OA): 1	0
2. Association of American Medical College	Gold OA: 1	0
3. Canadian Medical Education Journal	Gold OA: 1	0
4. Korea Health Personnel Licensing Examination Institute	Gold OA: 1	0
5. Society of Teachers of Family Medicine	Gold OA: 2	0
6. Shiraz University of Medical Sciences & Health Sciences	Gold OA: 2	75.00
7. Korean Society of Medical Education	Gold OA: 1	400.00
8. Health & Medical Publishing Group.	Gold OA: 1	438.00
9. Gesellschaft für Medizinische Ausbildung in the Association of Scientific Medical Societies	Gold OA: 1	856.00
10. Department of Public Health, Kaohsiung Medical University	Gold OA: 1	1,000.00
11. Ubiquity Press	Gold OA: 1	1,176.00
12. American Thoracic Society	Gold OA: 1	1,346.00
13. CCSP Press	Gold OA: 1	1,400.00
14. International Journal of Medical Education	Gold OA: 1	1,725.00
15. AMEE	Gold OA: 1	1,960.00
16. JMIR Publications Inc.	Gold OA: 1	1,985.00
17. SAGE Publications	Gold OA: 1	2,500.00
18. Dove Medical Press	Gold OA: 1	2,680.00
19. Wolters Kluwer	Hybrid: 4<br>Gold OA: 2	3,578.00
20. Elsevier	Hybrid: 7<br>Gold OA: 1	3,045.50
21. Springer	Hybrid: 4<br>Gold OA: 2	3,090.00
22. Frontiers Media	Gold OA: 1	3,295.00
23. Taylor & Francis	Hybrid: 6<br>Gold OA: 1	3,300.00
24. Wiley	Hybrid: 3	3,620.00

© 2024 Abdel-Razig S et al. *JAMA Network Open*

eTable 3. Discount and Waiver Policies of Health Professions Education Journals

**Journal**	**Policy**
Perspectives on Medical Education	If you would like to request an Article Processing Charge (APC) discount or waiver, this must be detailed in the cover letter during submission. All requests for waivers will be reviewed and decided separately. Waivers will only be considered if the first author is from LMI-Income Countries (LMIC) as defined by the World Bank: Angola; Algeria; Bangladesh; Benin; Bhutan; Bolivia; Cabo Verde; Cambodia; Cameroon; Comoros; Congo, Rep.; Côte d'Ivoire; Djibouti; Egypt, Arab Rep.; Eswatini; Ghana; Guinea; Haiti; Honduras; Jordan; India; Iran, Islamic Rep.; Kenya; Kiribati; Kyrgyz Republic; Lao PDR; Lebanon; Lesotho; Mauritania; Micronesia, Fed.Sts.; Mongolia; Morocco; Myanmar; Nepa; Nicaragua; Nigeria; Pakistan; Papua New Guinea; Philippines; Samoa; São Tomé and Principe; Senegal; Solomon Islands; Sri Lanka; Tanzania; Tajikistan; Timor-Leste; Tunisia; Ukraine; Uzbekistan; Vanuatu; Zambia; Zimbabwe.
International Journal of Medical Education	IJME offers waivers and discounts to corresponding authors based in low- and middle-income countries.
Journal of Medical Education and Curricular Development	Corresponding Authors who reside in the countries described by the Research4Life program (both Group A and Group B) publishing in one of Sage's gold open access journals will automatically receive a full APC waiver without needing to take any action. The waiver will be applied based on the Corresponding Author affiliation in the Sage Open Access Portal.
Academic Pathology	We automatically apply Article Publishing Charge waivers or discounts to those articles in gold open access journals for which all authors are based in a country eligible for the Research4Life program.<br><br>Our Online Author Communication System (OACS) ensures you are offered the lowest possible Article Publishing Charge to publish an article in your chosen journal. During submission you will be presented with a personalized OA Article Publishing Charge based on your individual context (your country, institutional affiliation, and any society membership for example) as well as considering the journal involved.
Medical Education Online	A 50% discount on the normal APC is also available to authors from countries defined by the World Bank as LMI income economies, who have a 2021 gross domestic product (GDP) of less than 200 billion US dollars.
BMC Medical Education	Routinely waive charges for authors from low-income countries.<br><br>Springer Nature offers APC waivers to papers whose corresponding authors are based in countries classified by the World Bank as low-income economies.
BMC Advances in Simulation	We routinely waive charges for authors from low-income countries. For other countries, article-processing charge waivers or discounts are granted

© 2024 Abdel-Razig S et al. *JAMA Network Open*

	on a case-by-case basis to authors with insufficient funds. Authors can request a waiver or discount during the submission process. For further details, see our article-processing charge page.

© 2024 Abdel-Razig S et al. *JAMA Network Open*
