# Supplementary material for: Open Access Publishing Metrics, Cost, and Impact in Health Professions Education Journals
Source: JAMA Netw Open. 2024 Oct 16;7(10):e2439932. doi: 10.1001/jamanetworkopen.2024.39932 (PMC11484459; doi:10.1001/jamanetworkopen.2024.39932)
Supplement: Supplement 2. — Data Sharing Statement [file jamanetwopen-e2439932-s002.pdf]

Data Sharing Statement

Abdel-Razig. Open Access Publishing Metrics, Cost, and Impact in Health Professions Education Journals. *JAMA Netw Open*. Published October 16, 2024.
doi:10.1001/jamanetworkopen.2024.39932

Data
Data available: Yes
Data types: Data (not involving human participants)
How to access data: please see Supplement 1
When available: With publication

Supporting Documents
Document types: Other (please specify)
Additional Information: eTable 1. Health Professions Education Journal Characteristics
eTable 2. Discount and Waiver Policies of Health Professions Education Journals
How to access documents: please see Supplement 1
When available: With publication

Additional Information
Who can access the data: All data is included in the manuscript or as supplementary material.
Types of analyses: All data is included in the manuscript or as supplementary material.
Mechanisms of data availability: NAAll data is included in the manuscript or as supplementary material.
